# *Aedes albopictus* is a competent vector of five arboviruses affecting human health, greater Paris, France, 2023

**DOI:** 10.2807/1560-7917.ES.2024.29.20.2400271

**Published:** 2024-05-16

**Authors:** Chloé Bohers, Marie Vazeille, Lydia Bernaoui, Luidji Pascalin, Kevin Meignan, Laurence Mousson, Georges Jakerian, Anaïs Karch, Xavier de Lamballerie, Anna-Bella Failloux

**Affiliations:** 1 Institut Pasteur, Université Paris Cité, Arboviruses and Insect Vectors, Paris, France; 2 Agence Régionale de Démoustication, Rosny-sous-Bois, France; 3 National Reference Center for Arboviruses, Inserm-IRBA, Marseille, France; 4 Unité des Virus Émergents (UVE: Aix-Marseille Univ, Università di Corsica, IRD 190, Inserm 1207, IRBA), Marseille, France

**Keywords:** *Aedes albopictus*, vector competence, arboviruses, France, risk assessment

## Abstract

*Aedes albopictus* collected in 2023 in the greater Paris area (Île-de-France) were experimentally able to transmit five arboviruses: West Nile virus from 3 days post-infection (dpi), chikungunya virus and Usutu virus from 7 dpi, dengue virus and Zika virus from 21 dpi. Given the growing number of imported dengue cases reported in early 2024 in France, surveillance of *Ae. albopictus* should be reinforced during the Paris Olympic Games in July, when many international visitors including from endemic countries are expected.

In 2023, nine sites of autochthonous dengue transmission were identified in mainland France, resulting in a total of 45 dengue infections. Three cases were detected in the greater Paris area (Île-de-France, Limeil-Brévannes) in September 2023 [1]. From 1 January to 19 April 2024, 1,679 imported dengue cases have been reported in France (13 times more than in 2023 during the same period) by the French public health agency ‘Santé Publique France’, with over 82% of cases returning from the French West Indies where an epidemic has been ongoing since mid-2023 [2]. In anticipation of the Olympic Games, which will take place in Paris from 26 July 2024 and bring together people from many nations around the world, we have estimated the vector competence of *Aedes albopictus* from department 92 (referred to herein as *Aedes albopictus* 92) for five arboviruses, chikungunya (CHIKV), dengue (DENV), Usutu (USUV), West Nile (WNV) and Zika (ZIKV).

## Assessing the vector competence

*Aedes albopictus* (F2–F3 generations) were originally collected in the department of Hauts de Seine (Île-de-France) in June 2023 from ovitraps placed in the cities of Colombes and Villeneuve-la-Garenne. Batches of 1-week-old female mosquitos were exposed to an infectious blood meal containing the viral suspension. Fed mosquitoes were sorted, supplied with 10% sucrose solution and incubated at 20 °C or 28 °C. We analysed mosquitoes at different days post-infection (dpi) depending on the virus (3, 7 and 14 dpi for CHIKV, USUV and WNV, and 7, 14 and 21 for DENV and ZIKV) to estimate three parameters describing the vector competence: (i) infection rate (IR) measuring the proportion of mosquitoes with an infected abdomen (including the midgut) among analysed mosquitoes, (ii) stepwise dissemination rate (SDR) corresponding to the proportion of mosquitoes with infected head and thorax among mosquitoes having an infected abdomen, which measures the ability of the virus to cross the midgut barrier and to disseminate into the mosquito’s internal cavity and (iii) stepwise transmission rate (STR) estimating the proportion of mosquitoes presenting virus in saliva among mosquitoes with infected head and thorax, which reflects the ability of the virus to invade the salivary glands, replicate and be excreted with the saliva delivered through the mosquito bite. Values of SDR and STR assign the relative importance of, respectively, the midgut and salivary glands as barriers to the progression of viral infection in the mosquito; the higher the value, the less effective the barrier is.

## *Aedes albopictus* can transmit typical *Aedes*-borne viruses CHIKV, DENV and ZIKV

Around 260 female mosquitoes were exposed to an infectious blood meal containing: (i) CHIKV 2010_1909 [3] at a titre of 10^7^ foci-forming units (ffu)/mL, (ii) DENV-1 2010_1806P France [4] at a titre of 10^7^ ffu/mL and (iii) ZIKV MRS OPY Martinique Pari 2015 [5] at a titre of 10^7^ tissue culture infectious dose 50% (TCID_50_)/mL. Engorged mosquitoes were only maintained at 28 °C [6].

For mosquitoes infected with CHIKV and DENV, samples were titrated by ffu on *Ae. albopictus* C6/36 cells [7]. After an incubation period at 28 °C of 3 days for CHIKV and 5 days for DENV, cells were stained using hyper-immune ascetic fluid specific to each virus as the primary antibody (provided by the French National Reference Center for Arboviruses) for CHIKV and Ms X dengue complex MAB 8705 (Millipore) for DENV. Alexa Fluor 488 goat anti-mouse IgG (Life Technologies) was used as the secondary antibody. For mosquitoes infected with ZIKV, samples were titrated by plaque-forming units (pfu) on Vero cells [7]. After a 7-day incubation period at 37 °C, cells were stained with a solution of safranine (0.5% in 10% formaldehyde and 10% ethanol). Presence of viral particles was assessed by detection of cytopathic effect.

**Figure 1:**
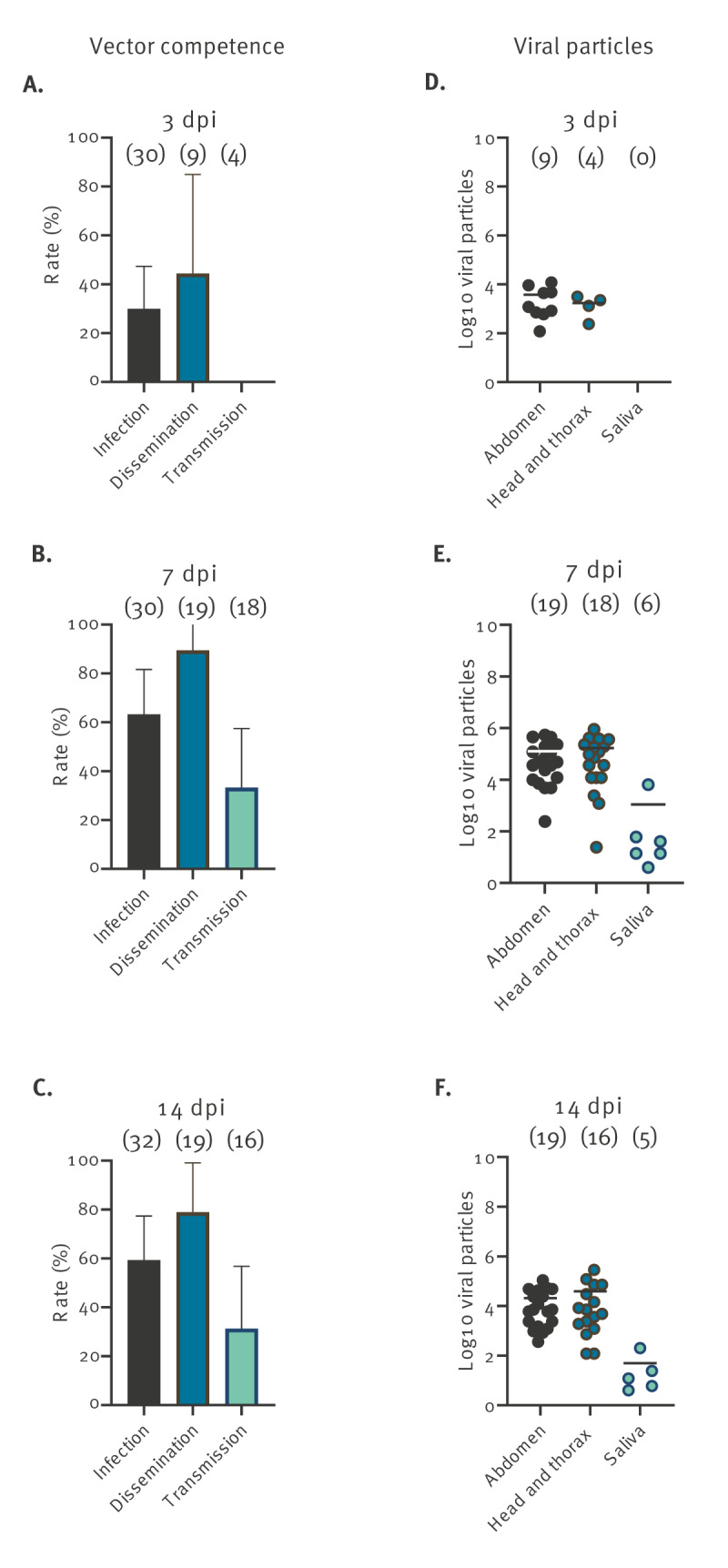
Vector competence and number of viral particles in the abdomen, head and thorax, and saliva of *Aedes albopictus* exposed to chikungunya virus, greater Paris, France, 2023 (n = 273)

For mosquitoes infected with CHIKV, viral infection (IR), stepwise dissemination (SDR) and stepwise transmission (STR) reached the highest values at 7 dpi, with 19 of 30 of mosquitoes having an infected midgut. Among these 19, 17 had an infected head and thorax, and of those, 6 had infectious saliva, meaning that the salivary glands played a stronger role as barrier to virus progression in the mosquito than the midgut (Figure 1B). Mosquitoes started to excrete viruses in saliva from 7 dpi with a mean of 1,088 viral particles (Figure 1E); the extrinsic incubation period (EIP) which is the duration between the ingestion and the excretion of the virus in mosquito saliva, is therefore 7 days for CHIKV.

dpi: days post-infection.

*Aedes albopictus* were exposed to an infectious blood meal containing chikungunya virus at a titre of 10^7^ foci-forming units/mL. Engorged mosquitoes were maintained at 28 °C until examination.

A–C. Batches of mosquitoes were processed to measure vector competence: (i) infection rate corresponding to the proportion of mosquitoes with an infected abdomen among the total number of examined mosquitoes, (ii) stepwise dissemination rate referring to the proportion of mosquitoes with infected head and thorax among mosquitoes with an infected abdomen and (iii) stepwise transmission rate representing the proportion of mosquitoes with infectious saliva among mosquitoes with infected head and thorax. The error bars show the 95% confidence intervals. The numbers of mosquitoes tested are in parentheses.

D–F. Abdomen, head and thorax, and saliva were processed for viral titration on mosquito cells. The solid bars represent the mean. The numbers of mosquitoes tested are in parentheses.

**Figure 2:**
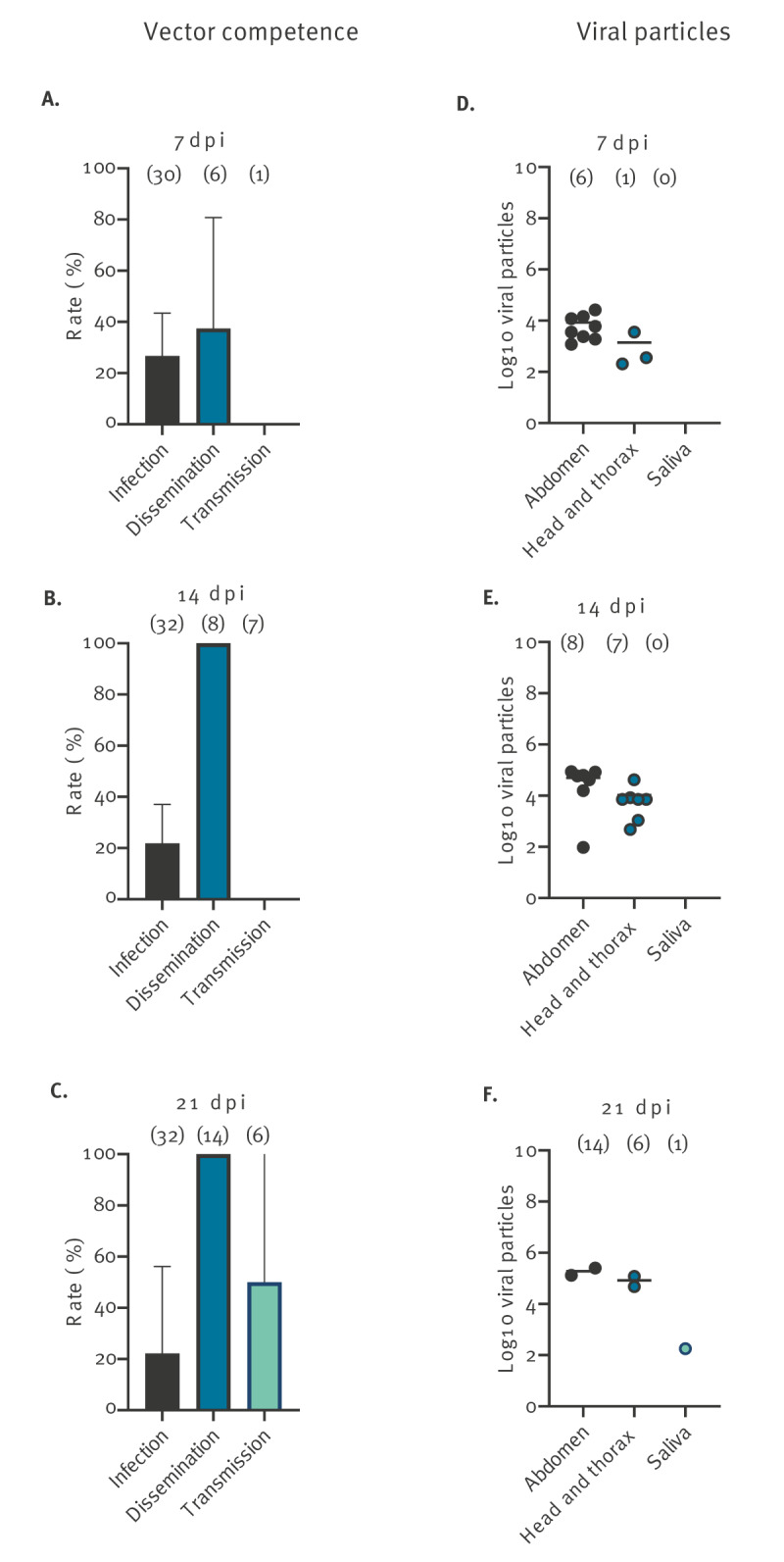
Vector competence and number of viral particles in the abdomen, head and thorax, and saliva of *Aedes albopictus* exposed to dengue virus, greater Paris, France, 2023 (n = 179)

For mosquitoes infected with DENV, stepwise dissemination and transmission rates reached the highest values at 21 dpi with SDR = 100%, and STR = 50% (Figure 2C), while the infection rate remained steady with IR = 21–26% suggesting that the critical step is midgut infection. Virus was detected in mosquito saliva at 21 dpi with 180 viral particles excreted by one female (Figure 2F). Therefore, EIP is 21 days for DENV.

dpi: days post-infection.

*Aedes albopictus* were exposed to an infectious blood meal containing dengue-1 virus at a titre of 10^7^ foci-forming units/mL. Engorged mosquitoes were maintained at 28 °C until examination.

A–C. Batches of mosquitoes were processed to measure vector competence: (i) infection rate corresponding to the proportion of mosquitoes with an infected abdomen among the total number of examined mosquitoes, (ii) stepwise dissemination rate referring to the proportion of mosquitoes with infected head and thorax among mosquitoes with an infected abdomen and (iii) stepwise transmission rate representing the proportion of mosquitoes with infectious saliva among mosquitoes with infected head and thorax. The error bars show the 95% confidence intervals. The numbers of mosquitoes tested are in parentheses.

D–F. Abdomen, head and thorax, and saliva were processed for viral titration on mosquito cells. The solid bars represent the mean. The numbers of mosquitoes tested are in parentheses.

**Figure 3:**
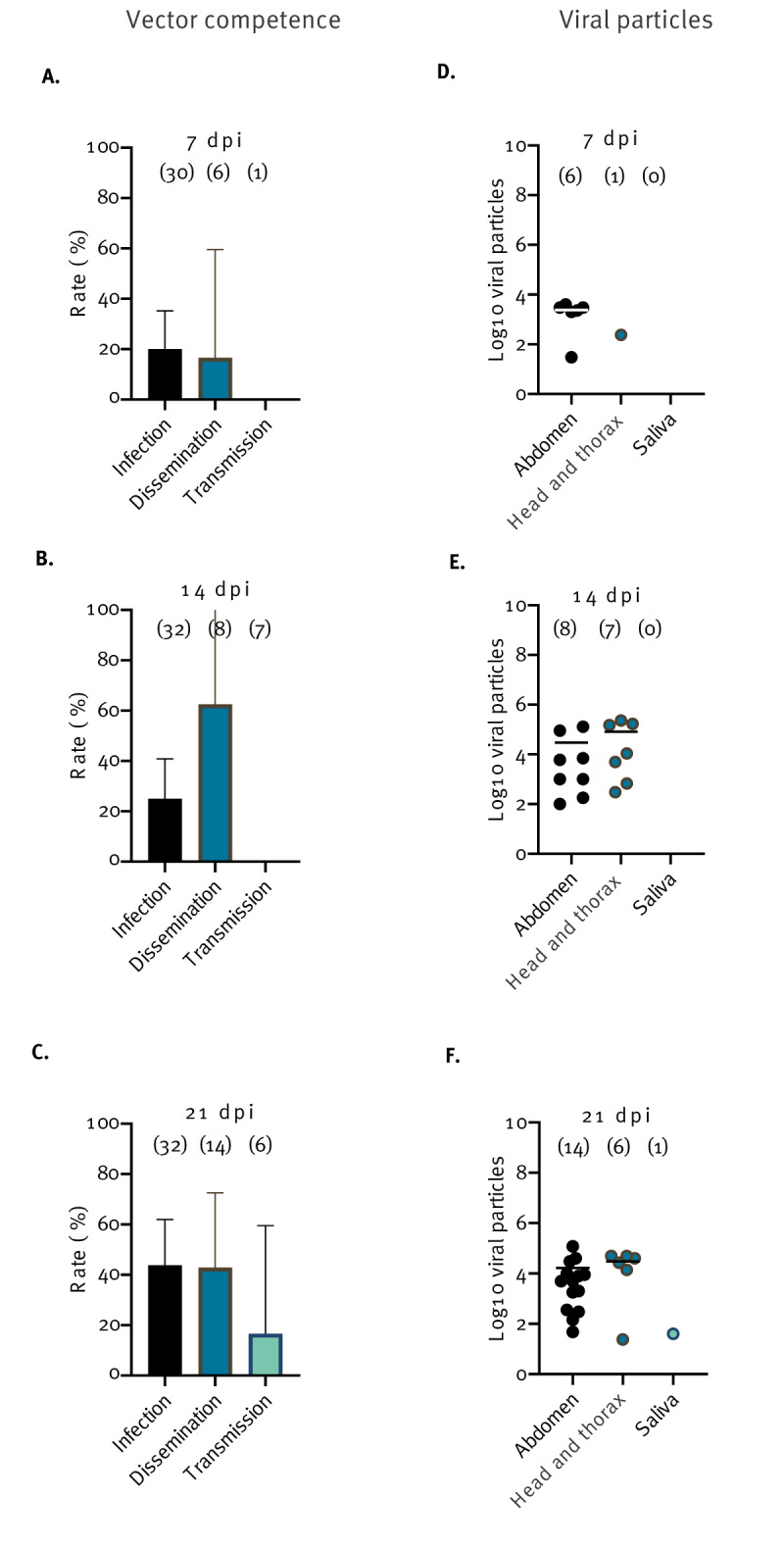
Vector competence and number of viral particles in the abdomen, head and thorax, and saliva of *Aedes albopictus* exposed to Zika virus, greater Paris, France, 2023 (n = 179)

For mosquitoes infected with ZIKV, viral infection was lower than 50%, varying from 20% at 7 dpi to 43.7% at 21 dpi (Figure 3A) while dissemination peaked at 14 dpi with SDR = 62.5% (Figure 3B) suggesting a minimal role of the midgut as barrier to virus dissemination. Transmission occurred only at 21 dpi with STR = 16.7% (Figure 3C) and 40 viral particles excreted by one female (Figure 3F). The EIP for ZIKV is therefore 21 days.

dpi: days post-infection.

*Aedes albopictus* were exposed to an infectious blood meal containing Zika virus at a titre of 10^7^ tissue culture infectious dose 50% (TCID_50_)/mL. Engorged mosquitoes were maintained at 28 °C until examination.

A–C. Batches of mosquitoes were processed to measure: (i) infection rate corresponding to the proportion of mosquitoes with an infected abdomen among the total number of examined mosquitoes, (ii) stepwise dissemination rate referring to the proportion of mosquitoes with infected head and thorax among mosquitoes with an infected abdomen and (iii) stepwise transmission rate representing the proportion of mosquitoes with infectious saliva among mosquitoes with infected head and thorax. The error bars show the 95% confidence intervals. The numbers of mosquitoes tested are in parentheses.

D–F. Abdomen, head and thorax, and saliva were processed for viral titration on mosquito cells. The solid bars represent the mean. The numbers of mosquitoes tested are in parentheses.

## *Aedes albopictus* is able to transmit two *Culex*-borne viruses at 28 °C but not at 20 °C

The *common* house mosquito *Culex pipiens* is the vector of WNV and USUV. Batches of 780 *Ae. albopictus* mosquitoes were exposed to an infectious blood meal containing: (i) WNV-1a 2000 Camargue [8] at a titre of 10^7^ pfu/mL and (ii) USUV-Haut Rhin 7315 Europa3 [9] (provided by Anses, Laboratoire de Santé Animale, UMR1161 Virology) at a titre of 10^7^ pfu/mL. Engorged mosquitoes were maintained at 20 °C or 28 °C. For WNV-infected mosquitoes, samples were titrated on Vero cells [10]. After 5 days at 37 °C, lytic plaques were counted after staining with a solution of safranine (0.5% in 10% formaldehyde and 20% ethanol). For USUV-infected mosquitoes, samples were titrated on C6/36 cells [10]. After 5 days at 28 °C, cells were fixed with 3.6% formaldehyde, washed and hybridised with anti-flavivirus monoclonal antibody (MAB10216, Millipore), and revealed by using a fluorescent-conjugated secondary antibody (A-11029, Life Technologies). Foci were counted under a fluorescent microscope.

**Figure 4:**
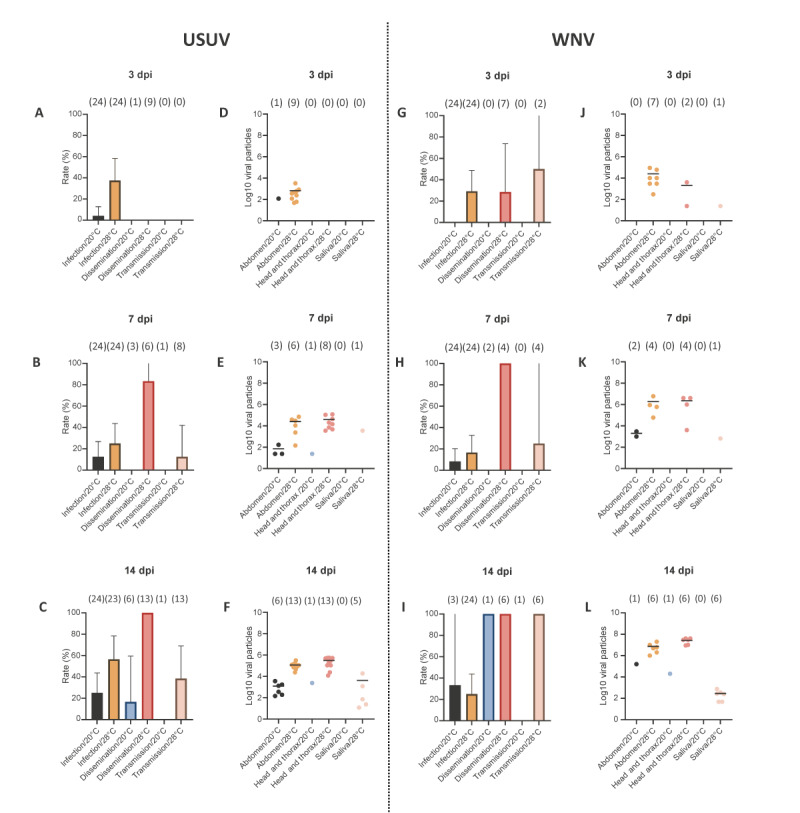
Vector competence and number of viral particles in the abdomen, head and thorax, and saliva of *Aedes albopictus* exposed to Usutu virus (n = 271) and West Nile virus (n = 197), greater Paris, France, 2023

For mosquitoes infected with USUV, IR and SDR peaked at 14 dpi when mosquitoes were incubated at 20 °C, with rates of 25% and 16.6%, respectively (Figure 4C). However, no transmission was observed regardless of the dpi. When incubated at 28 °C, infection, dissemination and transmission reached the highest values at 14 dpi (IR = 56.5%, SDR = 100%, and STR = 38.5%), revealing the more important role of the salivary glands as anatomical barriers to virus spreading in the mosquito than the midgut (Figure 4C). Mosquitoes started to excrete viral particles from 7 dpi with 3,600 viral particles excreted by one mosquito (Figure 4E), reaching a mean of 4,101 particles at 14 dpi (Figure 4F). The EIP for USUV was determined to be 7 days.

dpi: days post-infection; USUV: Usutu virus; WNV: West Nile virus.

*Aedes albopictus* were exposed to an infectious blood meal containing USUV (A–F) or WNV at a titre of 10^7^ pfu/mL (G–L). Engorged mosquitoes were maintained at 28 °C until examination.

A–C and G–I. Batches of mosquitoes were processed to measure: (i) infection rate corresponding to the proportion of mosquitoes with an infected abdomen among the total number of examined mosquitoes, (ii) stepwise dissemination rate referring to the proportion of mosquitoes with infected head and thorax among mosquitoes with an infected abdomen and (iii) stepwise transmission rate representing the proportion of mosquitoes with infectious saliva among mosquitoes with infected head and thorax. The error bars show the 95% confidence intervals. The numbers of mosquitoes tested are in parentheses.

D–F and J–L. Abdomen, head and thorax, and saliva were processed for viral titration on mosquito cells. The solid bars represent the mean. The numbers of mosquitoes tested are in parentheses.

For mosquitoes infected with WNV and incubated at 20 °C, IR and SDR culminated at 14 dpi, with rates of 33.3% and 100%, respectively (Figure 4I) without any transmission regardless of the dpi. When increasing to 28 °C, transmission (STR) reached 100% at 14 dpi (IR = 25% and SDR = 100%) showing a limited role of the midgut and salivary glands to virus spread in the mosquito (Figure 4I). Mosquitoes started to excrete viral particles from 3 dpi with 24 viral particles by a single mosquito (Figure 4J) followed by 660 particles by one mosquito at 7 dpi (Figure 4K) and a mean of 288 particles at 14 dpi (Figure 4L). The EIP for WNV is therefore 3 days.

## Discussion

After successfully establishing itself in mainland France in 2004 [11], *Ae. albopictus* was first detected in the region of Ile-de-France in 2015 (Val de Marne) [12]. *Aedes albopictus* 92 is competent to transmit WNV from 3 dpi, CHIKV and USUV from 7 dpi, and DENV and ZIKV from 21 dpi when incubated at 28 °C. As in many other host–parasite associations, the outcome of infection depends on the specific pairing of vector population and arbovirus genotype known as genotype-by-genotype (G x G) interactions [13], meaning that our estimation of vector competence is specific to *Ae. albopictus* 92. The vector competence is a component of the vectorial capacity which corresponds to the efficiency of a vector population to transmit a pathogen under natural conditions. To our knowledge, our study provides the first results on vector competence of *Aedes albopictus* 92 for five arboviruses.

Present in 78 French departments [2] and more than 20 European countries [14], *Ae. albopictus* 92 is thus able to sustain a local transmission of five arboviruses during the summer season (average temperature: 15–25°C). Autochthonous transmission of the three *Aedes*-borne viruses, CHIKV, DENV and ZIKV, relies on the introduction of imported cases from countries where these viruses are actively circulating. Viral particles in patients’ blood are detected 5–6 days after the bite of an infected mosquito. Viraemia lasts 5–9 days with a level depending on the virus, a mean of 10^9^ RNA copies/mL for CHIKV [15], 10^6.12^ cDNA copies/mL for DENV [16], and 10^5.36^ RNA copies/mL for ZIKV [17]. Our artificial feeding system provides 10^7–8^ viral particles/mL mixed with blood [7], which reflects patients’ viraemia and titres commonly used for assessing vector competence [18].

On the contrary, WNV and USUV require an enzootic cycle with sporadic spillovers to cause human cases. Birds are both the natural reservoir and amplifying host. In France, *Culex pipiens* is the main vector but *Ae. albopictus* is an experimentally competent mosquito [10]. While they are considered dead-end hosts, WNV-infected humans develop a mean viraemia of 10^4.4^ RNA copies/mL [19] which can be technically insufficient to infect mosquitoes [20]. However, we showed that *Ae. albopictus* 92 can transmit as early as 3 dpi, which should be considered with priority interest as ca 25% of infected people develop neurological complications [21]. USUV frequently co-circulates with WNV in European countries [22]. Causing significant mortalities in the European avifauna for 20 years, USUV causes encephalitis in humans, but pathogenesis is poorly understood [22]. While USUV and WNV are genetically, antigenically and epidemiologically closely related, they are transmitted differently by mosquitoes, including *Ae. albopictus*. 

Since 2006, from 1 May to 30 November each year, Santé Publique France coordinates the surveillance of chikungunya, dengue and Zika in the French departments, in relation with the Regional Health Agencies (ARS) and the National Reference Center of arboviruses. In conjunction with the upcoming summer Olympic Games, France expects to welcome ~11 million visitors, including 1.5 million from foreign countries, some of which are endemic for certain arboviruses. Reinforcing surveillance of *Ae. albopictus* should be a priority in 2024 in anticipation of this large international event.

## Conclusion

Local transmission of CHIKV, DENV, and ZIKV rely on imported cases, while WNV and USUV circulation depends on enzootic transmission. Therefore, the early detection of imported cases is critical to anticipate their possible autochthonous transmission. Based on our EIP values, vector control should be implemented in less than 1 week when CHIKV-infected people are detected, and less than 3 weeks when DENV- and ZIKV-infected cases are identified.
