# Authors’ correction for Euro Surveill. 2024;29(20)

**DOI:** 10.2807/1560-7917.ES.2024.29.21.240516c

**Published:** 2024-05-23

**Authors:** Eurosurveillance editorial team

**Affiliations:** 1 European Centre for Disease Prevention and Control (ECDC), Stockholm, Sweden

In the article entitled Aedes albopictus is a competent vector of five arboviruses affecting human health, greater Paris, France, 2023 by Bohers et al., published on 16 May 2024, a sentence in the Discussion incorrectly cited that ‘25% of infected people develop neurological complications’. This was corrected to ‘25% of infected people develop symptoms’ on 21 May 2024 at the request of the authors.

